# Editorial: Insights in DNA fragmentation

**DOI:** 10.3389/fendo.2025.1570774

**Published:** 2025-02-24

**Authors:** Jordi Ribas-Maynou

**Affiliations:** Unit of Cell Biology and Medical Genetics, Department of Cell Biology, Physiology and Immunology, Autonomous University of Barcelona, Bellaterra, Spain

**Keywords:** DNA fragmentation, sperm analysis, fertilization, ICSI, IVF

A comprehensive evaluation of sperm quality is required in order to decipher the specific contribution of paternal gametes to fertility rates. In this regard, achieving good sperm quality is essential to ensure the gamete’s ability to reach the fertilization site, undergo the physiological changes associated with capacitation, and penetrate the egg (1). During this process, sperm chromatin remains tightly packaged in protamines that confer extraordinary protection against genotoxic agents (2). However, once fertilized, protamines need to be replaced by histones and sperm chromatin must be able to form the paternal chromosomes without losing of DNA integrity. Sperm DNA damage has been identified for decades as a factor directly associated with male infertility, preventing pregnancy or its progression (3).

The present Research Topic includes three original papers and two reviews on the topic of DNA damage, providing new insights into our understanding of human fertility.

First, in the research by Fu et al., entitled “*Elevated sperm DNA fragmentation is correlated with an increased chromosomal aneuploidy rate of miscarried conceptus in women of advanced age undergoing fresh embryo transfer cycle*”, researchers retrospectively analyzed a cohort of IVF/ICSI patients who underwent miscarriage to investigate whether sperm DNA fragmentation evaluated using the Sperm Chromatin Structure Assay (SCSA) was associated with the early miscarriage. Their results suggest that paternal sperm DNA damage contributes to embryo aneuploidy rates, which is particularly relevant when maternal age is increased. In such cases, their study calculated that the risk of suffering a miscarriage is 5.76 times higher than in patients with low rates of DNA damage.

In the study entitled “*Artificial oocyte activation using Ca^2+^ ionophores following intracytoplasmic sperm injection for low fertilization rate*”, Akashi et al. addressed fertilization failure due to oocyte activation deficiency and evaluated whether treatment with Calcium ionophore could improve fertilization, pregnancy, and live birth rates in couples with previous fertilization failure. Their findings indicated that Ca^2+^ ionophores improved live birth rates compared to conventional ICSI, increasing fertilization rates, the number of good-quality cleavage-stage embryos, and blastocyst formation. Additionally, their subgroup analyses showed that younger patients might benefit more from this method.

Research in human male fertility is continuously exploring strategies to improve sperm quality for clinical application. In the study by Li et al., entitled “*Short-interval second ejaculation improves sperm quality, blastocyst formation in oligoasthenozoospermic males in ICSI cycles: a time-lapse sibling oocytes study*”, the authors evaluated whether a second ejaculation within a short interval could benefit sperm quality in oligoasthenoteratozoospermic patients undergoing ICSI. Their work on sibling oocytes demonstrated that sperm quality improved in terms of motility, morphology, and DNA damage compared to the first ejaculation, which translated into a higher rate of good-quality blastocysts. However, in their cohort, no statistically significant improvement in live birth rates was observed.

Finally, two reviews were accepted in this Research Topic.

First, the review by Romano et al. examined whether antioxidants or testicular sperm extraction are effective methods to improve fertility outcomes. To provide objective answers, they compiled results from several research papers obtained through a systematic search of related keywords. Their main findings suggested that, although antioxidants may be useful in some cases, scientific evidence is heterogeneous and prone to high risk of bias due to small sample sizes and methodological differences among studies. Regarding testicular sperm extraction, evidence suggests that testicular sperm may exhibit lower DNA damage than ejaculated sperm, and some studies support its use to enhance IVF success rates. However, available evidence remains limited and requires further validation.

Second, the meta-analysis by Lo Giudice et al. focused on sperm quality and objectively evaluated whether short ejaculatory abstinence can improve sperm parameters. They compared data from short abstinence (<2 days) and long abstinence (>2 days) and observed that shorter abstinence periods were associated with higher progressive motility and lower DNA damage. Additionally, a meta-regression analysis revealed an inverse relationship between abstinence duration and sperm DNA damage. Therefore, their meta-analysis suggests that shorter abstinence periods could be beneficial for patients undergoing assisted reproduction.

## References

[1] Delgado-Bermúdez A Yeste M Bonet S Pinart E. A review on the role of bicarbonate and proton transporters during sperm capacitation in mammals. Int J Mol Sci. (2022) 23:6333. doi: 10.3390/IJMS23116333 PMC9180951 35683013

[2] Ribas-Maynou J Nguyen H Wu H Ward WS. Functional aspects of sperm chromatin organization. Results Probl Cell Differ. (2022) 70:295–311. doi: 10.1007/978-3-031-06573-6_10 36348112 PMC9671218

[3] Lewis S Aitken R. DNA damage to spermatozoa has impacts on fertilization and pregnancy. Cell Tissue Res. (2005) 322:33–41. doi: 10.1007/S00441-005-1097-5 15912407

